# The Influence of SGLT2 Inhibitors on the Immune System and Inflammatory Processes in Kidney Transplantation

**DOI:** 10.3390/ijms27198547

**Published:** 2026-09-24

**Authors:** Andrés García-Sánchez, Pablo Ortíz-Lazareno, Leonardo Pazarín-Villaseñor, Elodia Nataly Díaz-de la Cruz, Alondra Mora-Jiménez, Jorge Andrade-Sierra, Ernesto Germán Cardona-Muñoz, Sylvia Elena Totsuka-Sutto, Luis Francisco Gómez-Hermosillo, Jorge Casillas-Moreno, Tannia Isabel Campos-Bayardo, Daniel Román-Rojas, Juan Antonio Garzón-Lamarque, Alejandra Guillermina Miranda-Díaz

**Affiliations:** 1 Department of Physiology, University Center of Health Sciences, University of Guadalajara, Guadalajara 44360, Jalisco, Mexico; andres_garciasanchez_3@hotmail.com (A.G.-S.); endiaz.delacruz@gmail.com (E.N.D.-d.l.C.); jorg_andrade@hotmail.com (J.A.-S.); cameg1@gmail.com (E.G.C.-M.); sylvia.totsuka@academicos.udg.mx (S.E.T.-S.); tanniaisabelcb@gmail.com (T.I.C.-B.); daniel.rrojas@academicos.udg.mx (D.R.-R.); juan.garzon9902@alumnos.udg.mx (J.A.G.-L.); 2 Western Biomedical Research Center, Mexican Social Security Institute, Guadalajara 44340, Jalisco, Mexico; pablolazareno@gmail.com (P.O.-L.); leopazarin@hotmail.com (L.P.-V.); 3 Department of Nutrigenomics, University Center of Health Services, University of Guadalajara, Guadalajara 44360, Jalisco, Mexico; alondra.mora2239@alumnos.udg.mx; 4 Departamento de Cirugía General, Hospital Civil de Guadalajara “Juan I Menchaca”, Guadalajara 44360, Jalisco, Mexico; luisgomez_53@hotmail.com (L.F.G.-H.); jcasillas_moreno@hotmail.com (J.C.-M.)

**Keywords:** kidney transplantation, innate and adaptive immunity, TLR2 and TLR4, SGLT2 inhibitors

## Abstract

Kidney transplantation (KT) remains the optimal therapy for end-stage kidney disease (ESKD), yet long-term graft survival is limited by unremitting allograft dysfunction and non-immune treatment toxicities. Sterile inflammation primarily drives graft failure. This inflammatory process releases damage-associated molecular patterns (DAMPs). DAMPs activate pattern recognition receptors (PRRs), such as Toll-like receptors (TLRs) 2 and 4, from proximal tubule epithelial cells, endothelial compartments, and infiltrating myeloid lineages. PRR signaling and the release of pro-inflammatory cytokines (IL-1β, IL-18, TNF-α) promote pro-inflammatory M1 macrophage polarization and accelerate interstitial fibrosis and tubular atrophy (IFTA). While maintenance immunosuppression prevents adaptive rejection, it causes severe metabolic, cardiovascular, and oncologic complications, including post-transplant diabetes mellitus, hypertension, and malignancies. Sodium-glucose cotransporter 2 (SGLT2) inhibitors (dapagliflozin, empagliflozin, canagliflozin) have emerged as novel disease-modifying, immunomodulatory adjuncts that act independently of glycemic control. SGLT2 inhibition reactivates tubuloglomerular feedback to relieve intraglomerular hypertension, blunts hyperfiltration, and reduces tubular transport workload. This alleviates cortical hypoxia, curtails mitochondrial ROS production, and activates AMP-activated protein kinase (AMPK) to suppress NF-κB and NLRP3 inflammasome assembly. SGLT2 inhibitors downregulate TLR expression, drive a phenotypic shift toward reparative M2 macrophages, reduce proteinuria, and provide sustained volume decongestion without triggering compensatory activation of the renin–angiotensin–aldosterone system. By targeting both upstream bioenergetic strain and downstream fibrotic remodeling (YAP/TAZ axis), SGLT2 inhibitors complement standard immunosuppression to protect allograft architecture, reduce cardiovascular morbidity, and improve long-term transplant outcomes.

## 1. Introduction

Kidney transplantation (KT) remains the gold standard intervention for restoring long-term survival and quality of life in patients with end-stage kidney disease (ESKD). Despite substantial advances in early allograft survival, progressive long-term graft loss remains a formidable clinical challenge. Extending allograft longevity and patient survival while minimizing systemic treatment toxicities is a primary goal in modern transplant medicine [1].

Overcoming this challenge requires addressing a continuum of non-immune, immunological, and metabolic hurdles. Long-term outcomes are increasingly constrained by an evolving donor landscape featuring extended-criteria donors, subclinical immune activation, early donor-specific antibody (DSA) formation, and accelerated organ senescence. Crucially, conventional immunosuppressive regimens, while essential to prevent adaptive rejection, increase the risk of diabetes mellitus, severe hypertension, opportunistic infections, and malignancy [2].

Unraveling the sterile inflammatory cascades triggered during organ procurement and implantation is paramount. From the initial insult of ischemia–reperfusion injury (IRI) and the activation of pattern recognition receptors (PRRs) to the downstream infiltration of pro-inflammatory M1 macrophages and dendritic cells (DCs), innate immune pathways serve as central drivers of delayed graft function (DGF) and progressive interstitial fibrosis and tubular atrophy (IFTA) [3].

This review synthesizes the pathophysiological mechanisms governing renal allograft injury and chronic dysfunction. We outline the key activators and amplifiers of sterile post-transplant inflammation, the cellular dynamics of intragraft myeloid populations, and the major non-immune complications of maintenance immunosuppression. Crucially, we highlight the emerging role of sodium-glucose cotransporter-2 (SGLT2) inhibitors as novel disease-modifying agents in KT. Operating through unique hemodynamic, bioenergetic, and immunomodulatory pathways, SGLT2 inhibitors offer a promising, complementary strategy to dampen sterile inflammation, alleviate tubular metabolic stress, and protect long-term allograft architecture [4,5].

### 1.1. Inflammation in Organ Transplantation

Evidence from clinical trials and experimental models confirms that inflammation largely dictates the long-term success or failure of a transplanted organ. Characterizing the distinct phases of post-transplant inflammation is essential for developing targeted therapies that ultimately safeguard patient outcomes and quality of life [4].

In KT, SGLT2 inhibitors have emerged as promising adjuncts. These agents exert robust anti-inflammatory, antifibrotic, and immunomodulatory effects that extend well beyond their primary glycemic actions. Rather than replacing standard immunosuppressive maintenance, SGLT2 inhibitors act via non-immune pathways, complementing conventional therapeutic regimens.

At the cellular level, they attenuate metabolic overload and tubulointerstitial hypoxia by blocking glucose and sodium reabsorption in the proximal tubule, thereby reducing intracellular ATP consumption and ROS generation. Furthermore, SGLT2 inhibition suppresses NLRP3 inflammasome activation, markedly decreasing downstream proinflammatory cytokines, particularly IL-1β, IL-18, TGF-β1, MCP-1 (CCL2), and NF-κB. By downregulating key profibrotic and inflammatory mediators these agents directly attenuate IFTA, a major driver of late graft failure. Finally, emerging preclinical and translational data indicate that SGLT2 inhibitors promote tissue repair by shifting the phenotype from proinflammatory M1 macrophages to tissue-healing M2 macrophages [5].

#### Ischemia–Reperfusion (IR) Injury

**Figure 1:**
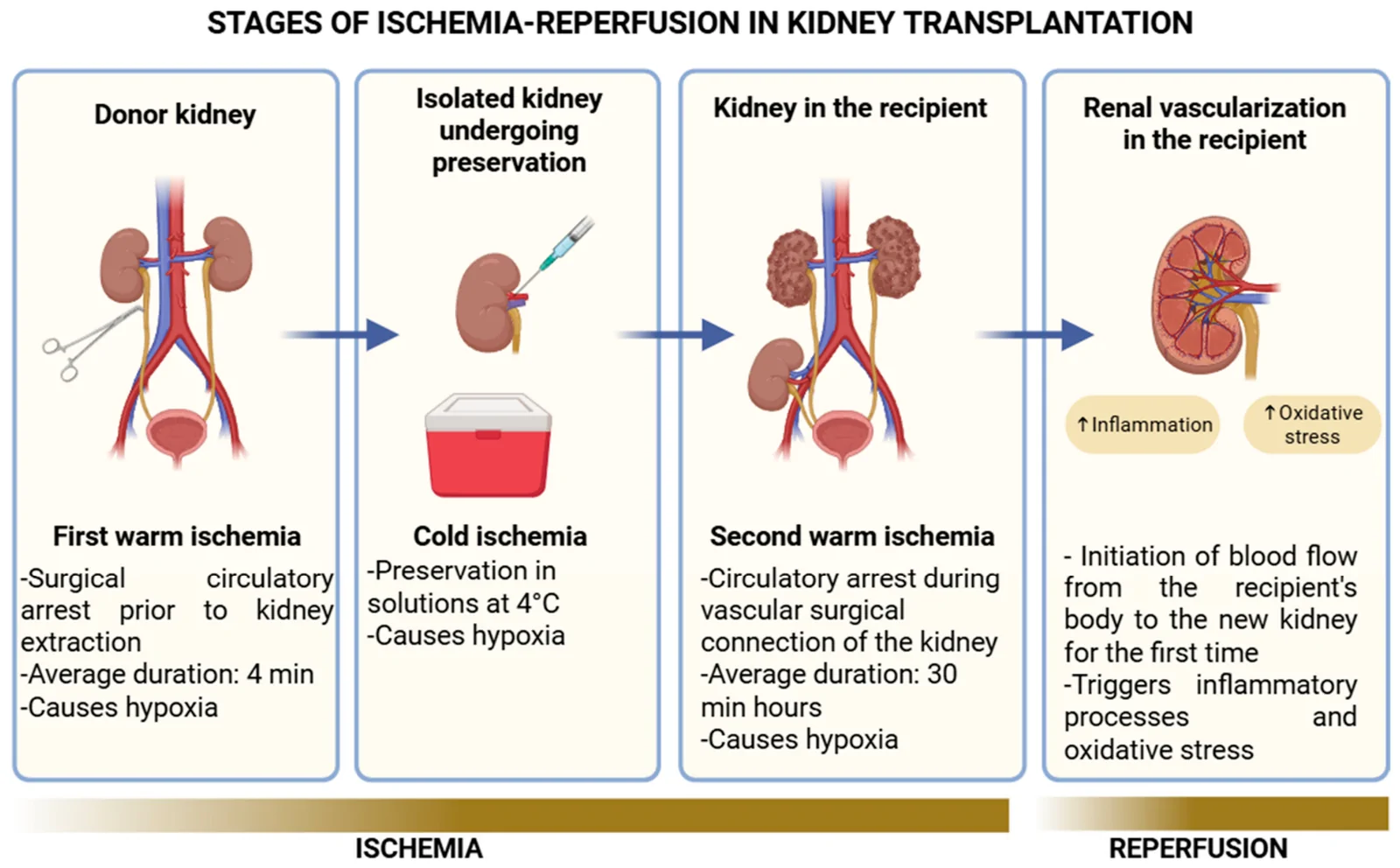
Stages of I/R injury in KT. Ischemia occurs during surgery to remove the kidney from the donor (first warm ischemia), during kidney preservation (cold ischemia), and during implantation into the recipient (second warm ischemia). Reperfusion occurs when blood first flows to the transplanted kidney. This blood flow triggers inflammatory and oxidative stress mechanisms that can damage the kidney [6]. The arrow indicates that reperfusion occurs after warm and cold ischemia. The blue and red colors represent venous (blue) and arterial (red) flow when blood flow is restore. Created in BioRender. Garcia, A. (2026) https://BioRender.com/430qxg9 (accessed on 17 August 2026).

I/R injury is the paradoxical damage that happens when blood flow returns after being cut off. In the first phase, the sudden loss of blood deprives cells of oxygen and causes toxic waste to build up quickly within the tissue [6] (Figure 1).

Tissue vulnerability to ischemia is highly cell-type dependent [6], with renal proximal tubular cells and hepatic Kupffer cells showing exceptional sensitivity to warm ischemic stress [7]. Although solid organ allografts can endure brief periods of warm ischemia or prolonged cold storage without immediate structural collapse, restoring blood flow paradoxically accelerates cellular damage. This process of kidney cellular damage is known as I/R injury [8]. In clinical transplantation, this phenomenon represents a major determinant of primary graft dysfunction and early graft failure [9]. Oxygen and nutrient deprivation during the ischemic phase destabilize the local microenvironment, creating a vulnerable state in which reoxygenation triggers a toxic response in both parenchymal and endothelial vascular compartments [10].

While I/R injury involves a complex web of pathophysiological pathways, robust experimental data pinpoint the innate immune response as the principal driver of the acute inflammatory cascade. Reperfusion triggers this inflammatory burst through complement activation, increased adhesion molecule expression, and rapid leukocyte recruitment [11]. Neutrophils, monocytes, and lymphocytes invade the organ tissue, amplifying parenchymal destruction via the synchronized release of ROS, chemokines, and pro-inflammatory cytokines [12].

SGLT2 inhibition has emerged as a promising strategy to mitigate I/R-induced damage and preserve tubular epithelial integrity. By inhibiting sodium and glucose reabsorption in the S1 and S2 segments of the proximal tubule, these agents reduce active transport demands, thereby lowering cellular oxygen consumption and relieving metabolic strain. Beyond metabolic support, SGLT2 inhibitors protect mitochondria and promote cell survival by shifting the apoptotic balance thereby concurrently curtailing ROS production and lipid peroxidation. Crucially, by suppressing the NLRP3 inflammasome axis and its downstream signaling cascade (IL-1β, IL-18, and TNF-α), this class of drugs blunts local leukocyte infiltration, protecting the allograft from severe tubulointerstitial damage [13].

### 1.2. Inflammation Activators and Amplifiers

I/R injury during KT inevitably causes parenchymal necrosis, thereby releasing endogenous danger-associated molecular patterns (DAMPs) into the extracellular space. The main DAMPs involved in parenchymal necrosis are HMGB1, heat shock proteins, S100 proteins, and extracellular/mitochondrial DNA. This leakage serves as an early alarm, inducing sterile inflammation by activating both the graft’s microvasculature and resident immune populations, including macrophages and dendritic cells (DCs) [14]. Concurrently, endothelial activation upregulates key adhesion molecules such as LFA-1 and VCAM-1, thereby facilitating the rapid recruitment of host neutrophils and circulating monocytes to the injury site [7]. These released DAMPs engage PRRs on tubular epithelial and innate immune cells. This interaction triggers downstream NF-κB and NLRP3 inflammasome cascades, driving early complications such as delayed graft function (DGF) and acute rejection. Consequently, characterizing these early innate immune ligands provides a compelling rationale for targeted pre-transplant therapies that can dampen graft inflammation, reduce systemic immunosuppression doses, and ultimately improve patient outcomes [15].

In this cascade, SGLT2 inhibitors potently disrupt innate immune amplification. By preserving mitochondrial integrity and attenuating ATP depletion in proximal tubule cells during I/R, these agents prevent necrotic cell death and directly limit the extracellular release of nuclear and mitochondrial DAMPs. At the receptor level, SGLT2 inhibition downregulates TLR4 and RAGE expression on tubular cells, desensitizing the tissue to circulating alarmins generated during organ procurement and reperfusion. Furthermore, by activating AMPK and suppressing ROS production, SGLT2 inhibitors halt the DAMP-TLR-driven assembly of the NLRP3 inflammasome, blocking the maturation of IL-1β and IL-18. Finally, this intervention reduces DAMP-induced secretion of chemokines such as MCP-1 (CCL2) and ICAM-1, thereby limiting infiltration of pro-inflammatory M1 macrophages into the graft interstitium [15].

### 1.3. Inflammation and Innate Immunity

Resident immune cells constitute the recipient’s first line of defense, rapidly sensing exogenous and endogenous signals to unleash a spectrum of effector molecules [8]. PRRs enable tissue macrophages, dendritic cells, and other cells of myeloid lineage to identify both PAMPs and DAMPs [16,17]. Upon activation, PRRs across five main classes (TLRs, CLRs, NLRs, RLRs, and SRs) initiate signaling through pathways such as NF-κB, inducing cytokine and interferon release, neutrophil adhesion to the graft microvasculature, and phagocytic responses [16].

Beyond clearing cellular debris, these innate activation pathways can paradoxically amplify ischemic tissue destruction or trigger programmed cell death [14]. Furthermore, once the initial inflammatory surge subsides, activated DCs and macrophages migrate to draining lymph nodes, bridging innate danger sensing with donor-specific adaptive immune responses [17]. Given their pivotal role in driving graft injury, PRRs have emerged as primary therapeutic targets to mitigate sterile post-transplant inflammation [18].

SGLT2 inhibitors (dapagliflozin, empagliflozin, and canagliflozin) act as novel immunomodulators, suppressing sterile innate signaling independently of their glucose-lowering actions. By metabolically reprogramming immune and renal tubular cells, these agents alter core PRR cascades, downregulating TLR2, TLR4, and RAGE expression in macrophages and epithelial cells and attenuating responsiveness to released DAMPs. At the intracellular level, elevated AMP/ATP ratios induced by SGLT2 inhibition activate AMP-activated protein kinase (AMPK), which directly represses NF-κB transcription, thereby reducing key chemoattractants (MCP-1/CCL2) and vascular adhesion markers (ICAM-1, VCAM-1) [19].

At the same time, this drug class preserves mitochondrial integrity and curtails ROS generation, blunting the NLRP3/ASC/Caspase-1 axis and preventing IL-1β and IL-18 maturation [20,21,22,23].

Beyond cytokine suppression, SGLT2 inhibition drives a phenotypic shift in infiltrating macrophages from a pro-inflammatory M1 state (CD86+, producing TNF-α and IL-6) toward a reparative M2 profile (CD206+, IL-10+), while limiting neutrophil extracellular trap (NET) formation and myeloperoxidase (MPO) activity [24]. Together, these actions curb acute tissue destruction and promote graft preservation.

### 1.4. The Inflammasome

As an intracellular multimeric complex, the inflammasome functions as a central sensor of cellular stress, assembling in response to danger signals such as PAMPs, DAMPs, and upstream TLR activation. Once triggered by I/R injury, calcineurin inhibitor toxicity, or metabolic overload, the sensor protein (such as NLRP3) recruits pro-caspase-1 via the adaptor protein ASC (encoded by *PYCARD*). This scaffolding platform enables proteolytic cleavage of caspase-1, thereby driving the maturation and secretion of potent pro-inflammatory cytokines, specifically IL-1β and IL-18, while orchestrating gasdermin D-mediated pyroptosis [25]. The pathogenic relevance of this pathway in transplantation is well documented; for instance, murine cardiac allograft models demonstrate a marked post-transplant upregulation of both *PYCARD* and IL-1β strictly within allogeneic grafts, a response absent in syngeneic controls. Despite these insights, the precise translational mechanisms governing inflammasome activation across different organ allografts warrant continued investigation [25].

SGLT2 inhibitors robustly suppress the NLRP3 inflammasome cascade in the renal graft through dual metabolic and organellar mechanisms. First, at the transcriptional level, SGLT2 inhibition elevates the intracellular AMP/ATP ratio, thereby activating AMP-activated protein kinase (AMPK). Active AMPK directly represses NF-κB signaling, blunting the initial priming step required for transcription of NLRP3 and pro-IL-1β. Second, at the organellar level, these agents preserve mitochondrial membrane potential, promote quality control via PINK1/Parkin-mediated mitophagy, and drastically reduce mitochondrial ROS generation. By halting both the priming and activation phases of the inflammasome, SGLT2 inhibitors protect tubular epithelial cells from pyroptotic cell death and limit sterile inflammation during allograft I/R injury [12].

### 1.5. Innate Immunity Receptors in the Kidney

**Figure 2:**
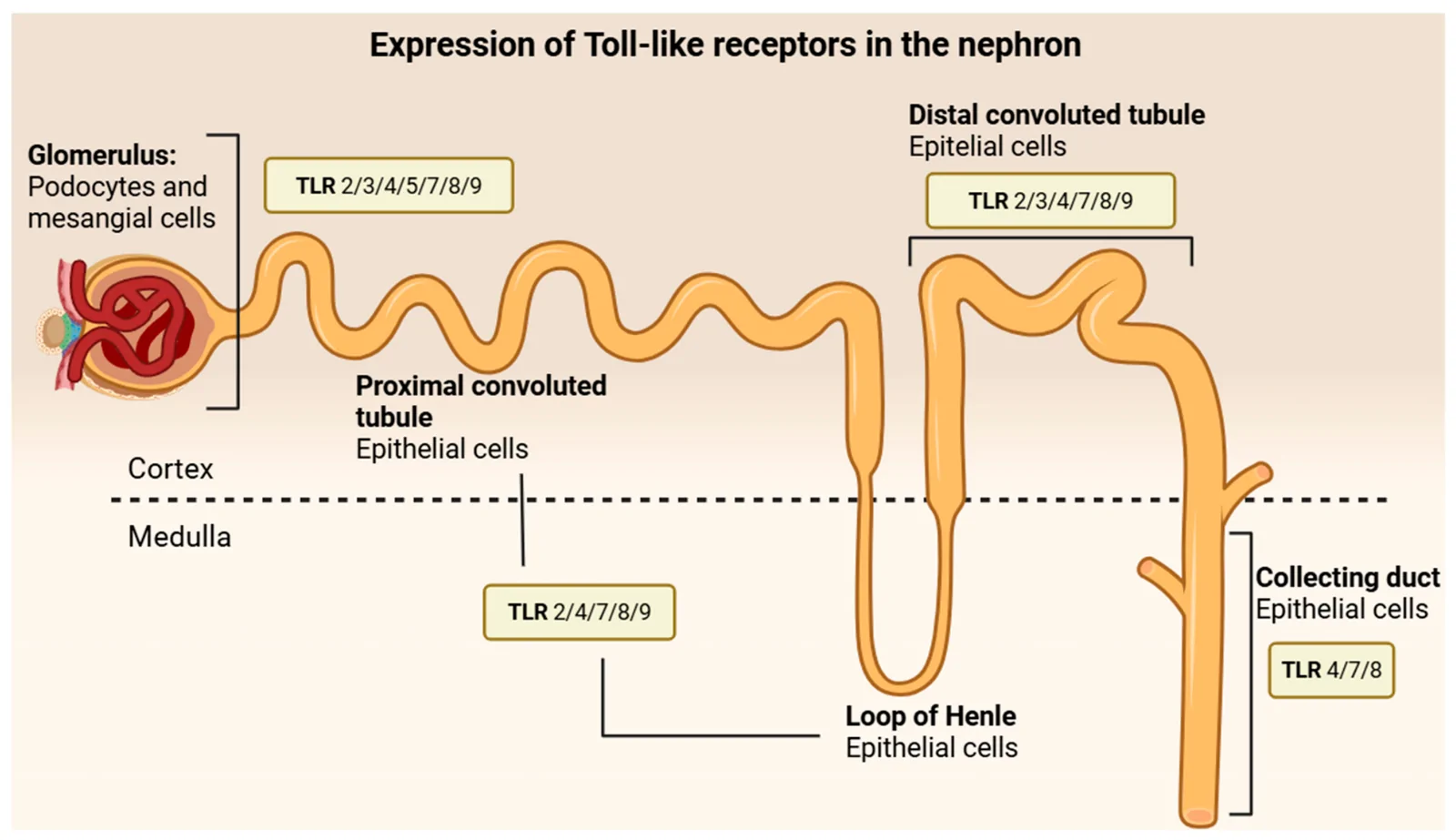
Expression of toll-like receptors in the nephron. Expression of Toll-like receptors in the nephron. The main cells participating in the nephron’s immune response via TLRs are tubular epithelial cells, podocytes, and mesangial cells [27,28]. Created in BioRender. Garcia, A. (2026) https://BioRender.com/wg45ff4 (accessed on 17 August 2026).

Within the renal architecture, PRRs are constitutively expressed across both parenchymal and infiltrating immune cell populations in human and rodent models. Murine and human studies confirm that these receptors display segment-specific differential expression along the nephron. Specifically, proximal tubular epithelial cells express a broad repertoire of TLRs, including TLR1–4, TLR6, and TLR9, whereas endothelial cells, mesangial cells, and podocytes prominently feature TLR2, TLR3, and TLR4 (Figure 2). In contrast, TLR5 and TLR11 localize predominantly to the distal tubule segments and the urothelium [26].

From a clinical and translational perspective, this compartmentalized expression directly influences allograft outcomes. For instance, elevated renal expression of TLR7 and TLR9 has been implicated in the pathogenesis of glomerulonephritis and tubulointerstitial inflammation [27], while specific TLR3 gene polymorphisms have been shown to correlate with an increased risk of delayed graft function (DGF) [28]. Notably, TLR4 is markedly overexpressed in allografts from deceased donors [29] and is a primary driver of I/R injury [30]. Demonstrating its immunogenic relevance, recipients or donors harboring loss-of-function TLR4 gene polymorphisms experience significantly fewer episodes of acute rejection, underscoring TLR4 as a crucial therapeutic target [30].

SGLT2 inhibitors directly and indirectly downregulate key innate immune receptors (TLR2, TLR4, and associated PRRs) on proximal tubular epithelial cells, vascular endothelium, and infiltrating myeloid-lineage cells. By modulating these receptor axes, SGLT2 inhibition blunts cellular responsiveness to endogenous danger signals, protecting the renal allograft from sterile post-transplant inflammation, I/R injury, and immunogenic tissue damage [31].

### 1.6. Immune Cells Involved in the Inflammatory Response and SGLT2 Modulation

The infiltration and activation of local leukocyte populations further aggravate the primary ischemic insult within the kidney. Activated neutrophils are among the earliest responders, discharging proteases, ROS, and pro-inflammatory mediators that compromise microvascular integrity and disrupt the structural barrier of renal tubular epithelial and endothelial cells [27]. The adhesion molecules ICAM-1, selectins, and the CD11/CD18 and integrin complex coordinate this extravasation and tissue recruitment [32].

In parallel with neutrophil influx, macrophages and dendritic cells (DCs) accumulate within the tissue, serving as central sentinels for innate danger sensing that bridge early injury and adaptive immunity [33]. Characterized by high basal expression of PRRs, DCs become activated following I/R injury. Once primed, these professional antigen-presenting cells amplify inflammation directly via T-cell engagement and indirectly through the release of pro-inflammatory cytokines [34]. Experimental models demonstrate that preventing DC maturation exerts a therapeutic immunosuppressive effect on ischemic injury [35]. However, depending on their activation state, DCs can also enforce immune tolerance and resolve inflammation [36].

Other lymphocyte subsets play pivotal, divergent roles during reperfusion. Natural killer T (NKT) cells expand rapidly within the kidney, releasing IFN-γ, TNF-α, IL-4, and IL-1β. Depleting NKT cells attenuates neutrophil influx and blunts renal damage, underscoring their role in orchestrating regulation [37]. Conversely, regulatory T (Treg) cells act as essential brakes on innate inflammation, homing to injured tissue to suppress damage by releasing IL-10 [38]. Finally, while B lymphocytes contribute to tissue recovery in some models, intragraft infiltration of mature B cells in renal transplantation correlates with reduced long-term graft survival. Recent evidence indicates that I/R-induced innate immune activation drives B cells to produce pathogenic antibodies, whereas B-cell deficiency mitigates ischemic injury and fosters tubular regeneration [39].

While KT remains the gold standard for restoring survival and quality of life in ESKD, long-term allograft longevity remains constrained by ischemic tissue damage sustained during organ retrieval [25]. Graft ischemia initiates a sterile inflammatory cascade driven by DAMP release, which engages PRRs across tubular epithelial cells, interstitial DCs, and the vascular endothelium. Intercepting PRR-mediated innate immune activation during this phase offers a dual benefit: dampening early cell death pathways and blunting downstream host inflammatory responses [40].

SGLT2 inhibitors (dapagliflozin, empagliflozin, canagliflozin) emerge as novel immunomodulators that target diverse leukocyte populations independently of their glucose-lowering effects [41].

## 2. Dendritic Cells and Macrophages in Kidney Transplantation

Although KT remains the definitive therapy for ESKD, long-term graft survival depends on continuous immunosuppression. Unraveling the precise mechanisms of allograft rejection is therefore critical to developing targeted strategies that safeguard graft longevity while minimizing the systemic toxicity of chronic immunosuppressive regimens [42]. Within the allograft microenvironment, DCs and macrophages constitute the primary myeloid orchestrators of innate surveillance, antigen presentation, sterile inflammation, and progressive tissue remodeling.

While DCs primarily coordinate antigen presentation to direct either T- and B-cell effector activation or immunological tolerance, macrophages function as classical innate effectors dedicated to phagocytic clearance and tissue remodeling [43]. In the renal microenvironment, the phenotypic and functional boundaries between these lineages often overlap because of shared surface markers and plastic effector roles. Rodent and human studies confirm that DCs reside throughout the interstitium and glomeruli, driving chronic cell-mediated inflammatory cascades in conditions like glomerulonephritis, lupus nephritis, and ischemia-induced acute kidney injury (AKI) [44].

Resident macrophages expand markedly during renal pathology, occupying a spectrum anchored by two classic functional states. Classical M1 macrophages (CD86+), induced by LPS or IFN-γ, secrete pro-inflammatory cytokines (TNF-α, IL-6, MCP-1) to coordinate early defense [45]. Conversely, alternatively activated M2 macrophages (CD206+, Arg-1+) exert anti-inflammatory and tissue-repairing actions via IL-10 release [46]. The M2 lineage comprises three functional subsets: M2a (wound-healing, primed by IL-4/IL-13), M2b (immunoregulatory, driven by immune complexes), and M2c (regulatory, dedicated to immunosuppression and matrix remodeling) [47].

Following acute ischemic or toxic insult, infiltrating monocytes differentiate into pro-inflammatory M1 macrophages under the influence of neutrophils, NK cells, and Th1/Th17 effectors. If the primary damage is severe or inflammation persists, macrophages become the main drivers of chronic kidney disease and allograft fibrosis. Macrophages secrete profibrotic mediators (TGF-β1, FGF-2, and PDGF) that stimulate myofibroblast proliferation and extracellular matrix deposition. Furthermore, macrophages synthesize collagen directly, undergo a macrophage-to-myofibroblast transition (MMT), and disrupt the peritubular microvasculature, thereby exacerbating local hypoxia [48,49,50].

SGLT2 inhibitors act as potent immunomodulatory agents that can directly and indirectly reprogram intragraft myeloid cells: SGLT2 inhibition significantly reduces chemoattractant signals (MCP-1/CCL2) and endothelial adhesion markers, thereby curbing monocyte recruitment into the graft interstitium during I/R and acute rejection [51]. SGLT2 inhibition reduces intracellular ROS generation and preserves mitochondrial membrane potential in macrophages, thereby preventing NLRP3 inflammasome assembly and blunting the secretion of mature IL-1β and IL-18. DCs exposed to SGLT2 inhibitors exhibit reduced expression of co-stimulatory molecules and lower secretion of IL-12 and IL-23. This blunted maturation limits the differentiation of naive recipient T cells into pathogenic Th1 and Th17 subpopulations within the allograft [51].

### 2.1. Toll-like Receptors

**Figure 3:**
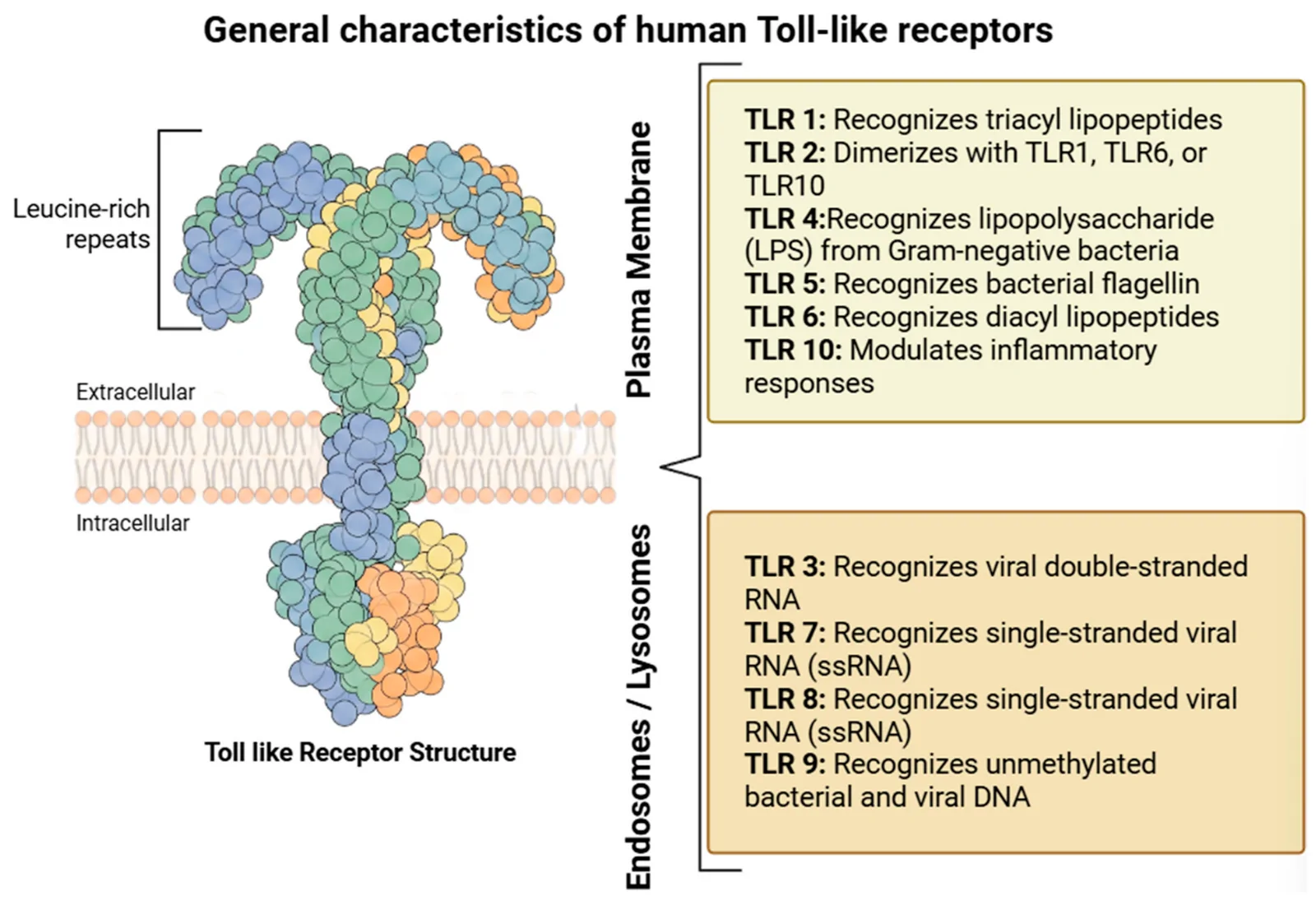
General characteristics and subcellular localization of human Toll-like receptors. Human TLRs are categorized by their surface or endolysosomal localization, each specialized in recognizing specific microbial antigens or tissue-derived DAMPs. (Created in BioRender. Garcia, A., 2026) https://BioRender.com/ma10qxj (accessed on 17 August 2026).

TLRs are the most extensively characterized family of PRRs that can sense both exogenous PAMPs and endogenous danger signals [51]. Structurally, these transmembrane proteins feature an extracellular leucine-rich repeat domain responsible for ligand binding, a single transmembrane domain, and a conserved intracellular Toll/interleukin-1 receptor (TIR) homology domain. Of the 13 mammalian TLRs identified to date, 10 are functionally expressed in humans, displaying distinct subcellular localizations: TLR1, TLR2, TLR4, TLR5, TLR6, and TLR10 reside on the cell membrane to monitor the extracellular space, whereas TLR3, TLR7, TLR8, and TLR9 localize to endosomal and lysosomal membranes to detect nucleic acids (Figure 3) [52,53].

Upon ligand engagement, TLRs recruit cytosolic adaptor proteins via their TIR domains to initiate intracellular signaling cascades [54,55]. Most TLRs signal through the MyD88-dependent pathway, driving nuclear translocation of NF-κB and AP-1 and inducing a robust pro-inflammatory secretome, including TNF-α and IL-6 [56]. Additionally, receptors such as TLR4 engage a MyD88-independent, TRIF-dependent branch that triggers type I interferon expression and distinct transcriptional profiles [57]. In renal sterile inflammation and ischemia–reperfusion, this persistent TLR activation drives cellular aging, oxidative stress, and progressive tissue loss [58].

SGLT2 inhibitors act as potent upstream and downstream disruptors of the TLR signaling axis, mitigating sterile inflammation, oxidative stress, and tubulointerstitial fibrosis across renal epithelial, endothelial, and infiltrating myeloid lineages: SGLT2 inhibition activates AMP-activated protein kinase (AMPK), which directly represses the MyD88-dependent NF-κB signaling cascade downstream of TLR2 and TLR4. These agents preserve cellular energy states and mitochondrial integrity during injury, thereby curbing the extracellular release of endogenous DAMPs that otherwise act as primary activating ligands for TLRs. SGLT2 inhibitors suppress both the gene expression and cell-surface density of TLR2 and TLR4 in renal proximal tubular epithelial cells, vascular endothelium, and tissue-resident macrophages, desensitizing these compartments to circulating danger signals [59].

### 2.2. TLRs in Human Kidney Disease

**Figure 4:**
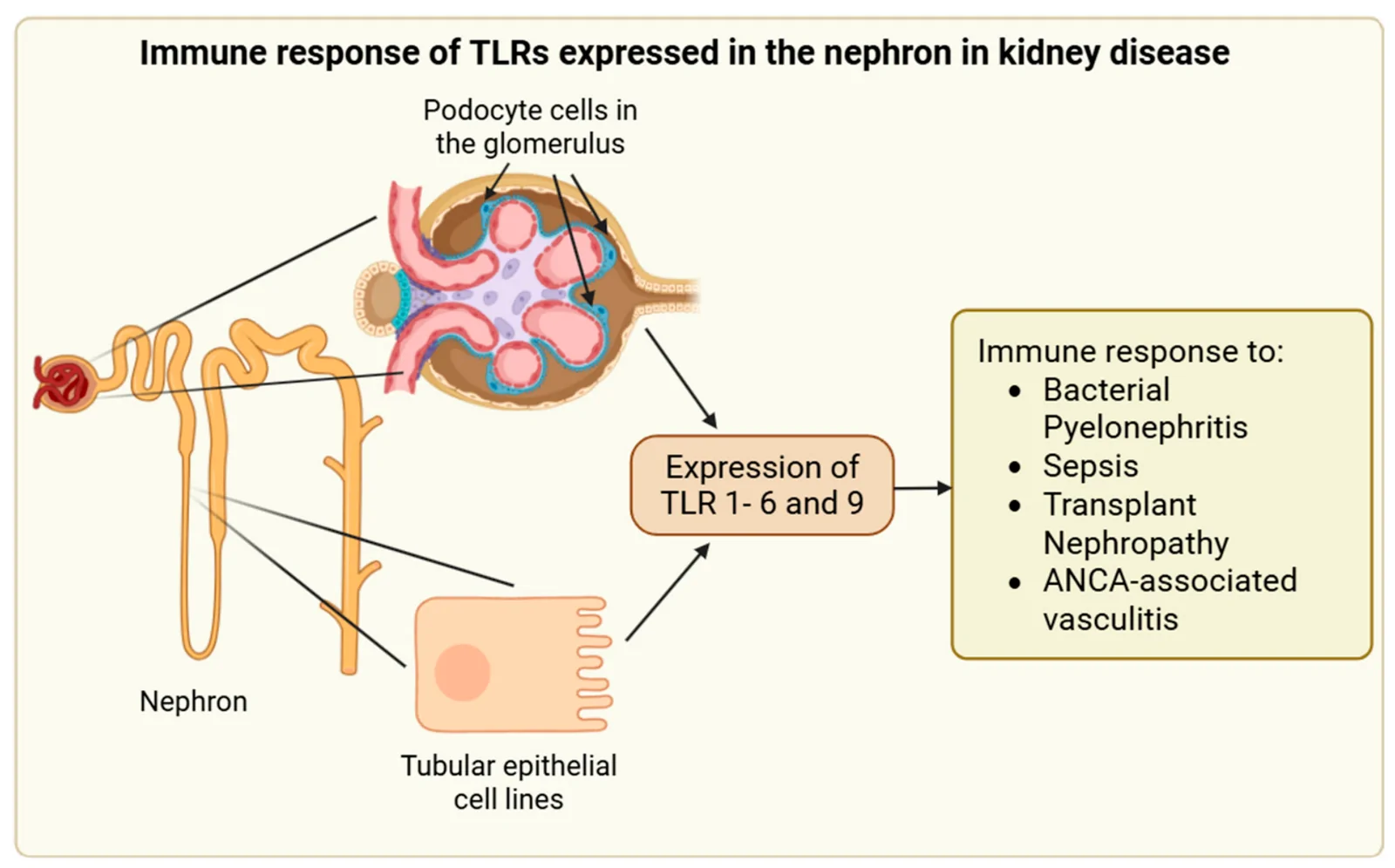
Immune response mediated by nephron-expressed TLRs in kidney disease. Immune response mediated by nephron-expressed TLRs in kidney disease. TLRs expressed on glomerular and tubular epithelial cells drive local inflammatory signaling in response to bacterial pyelonephritis, sepsis, transplant nephropathy, and ANCA-associated vasculitis. TLR, Toll-like receptor; ANCA, antineutrophil cytoplasmic antibodies [59,60,61]. (Created in BioRender. Garcia, A., 2026) https://BioRender.com/zic9r0f (accessed on 17 August 2026).

Immortalized human podocytes express functional transcripts for TLR1–6 and TLR9 [57], while human tubular epithelial cells constitutively feature TLR1–4 and TLR6 (Figure 4). This widespread expression in the glomerular and tubular compartments highlights their active role in orchestrating innate immune cascades across diverse tubulointerstitial pathologies, including bacterial pyelonephritis, sepsis-induced acute kidney injury, and transplant nephropathy [58]. In the proximal tubule, TLR expression is dynamically upregulated under pathological stress largely driven by high-mobility group box 1 (HMGB1), a nuclear protein that acts as a potent endogenous alarm trigger [59].

In glomerular endothelial cells and podocytes, elevated TLR2 and TLR4 levels are prominent hallmarks of ANCA-associated vasculitis [60]. In kidney transplantation, upregulation of the MyD88 gene within graft tissue and increased TLR4 mRNA expression in peripheral blood mononuclear cells (PBMCs) serve as clinical indicators of cell-mediated rejection [61] and overall graft dysfunction [62].

TLRs act as central sensors of metabolic and sterile cellular stress. SGLT2 inhibitors (empagliflozin, dapagliflozin, canagliflozin) directly and indirectly attenuate these intrarenal TLR pathways, dampening tubulointerstitial inflammation and delaying progressive allograft fibrosis.

SGLT2 inhibitors significantly downregulate TLR4 gene expression and suppress the nuclear binding of NF-κB and AP-1 transcription factors in proximal tubule cells. These agents preserve mitochondrial function, reduce intracellular ROS, and alleviate tubular hypoxia, thereby diminishing DAMP shedding and halting the self-perpetuating TLR activation loop. SGLT2 inhibition suppresses the intrarenal chemokine axis, blunting myeloid cell recruitment and shifting intrarenal macrophages from a TLR4-driven pro-inflammatory M1 phenotype toward a tissue-repairing M2 state. Functionally, this class of drugs reduces downstream release of pro-inflammatory cytokines (IL-6, IL-1β, IL-18) and key profibrotic mediators (TGF-β1, Collagen IV) in human tubule cells, preserving renal architecture [63].

### 2.3. Role of TLRs in Kidney I/R Injury

**Figure 5:**
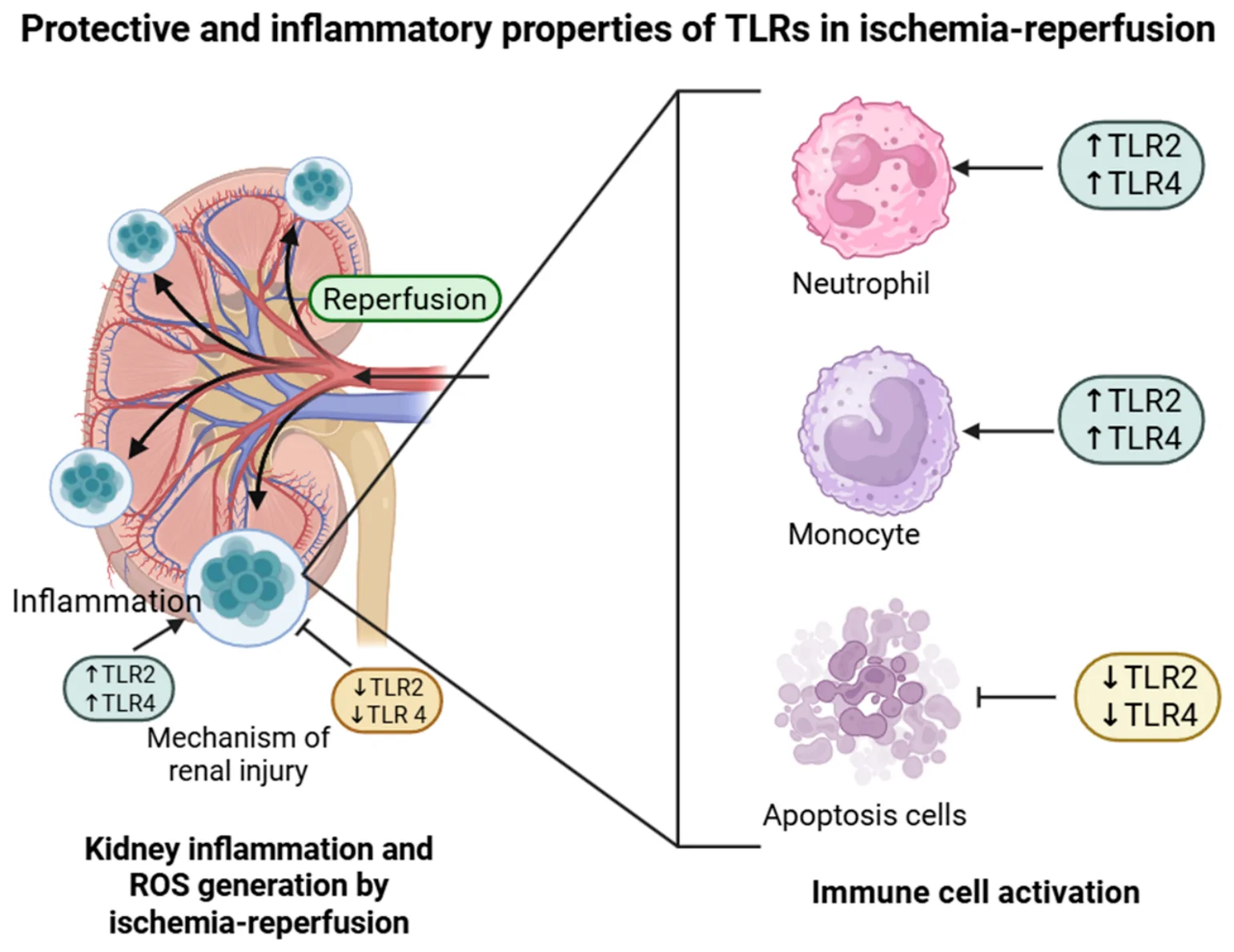
Protective and inflammatory properties of TLRs in I/R injury. TLR2 and TLR4 are the primary Toll-like receptors studied in renal I/R injury mechanisms. TLR2/4 signaling is linked to the activation of monocyte- and neutrophil-mediated inflammatory processes during I/R. Depleting TLR2/4 protects against renal injury by attenuating inflammatory processes in animal models [70,71,72]. Created in BioRender. Garcia, A. (2026) https://BioRender.com/a2oeij9 (accessed on 17 August 2026).

I/R injury is a frequent complication in critical clinical settings such as severe trauma and KT. Although human renal tissue expresses the full repertoire of TLRs, TLR2 and TLR4 stand out as central mediators of I/R-induced tissue damage (Figure 5). Under physiological conditions, both receptors exhibit basal expression along the nephron—particularly in the proximal and distal tubules, the thin limb of the loop of Henle, and the collecting ducts—which increases individually following an ischemic event [63].

Functional studies using knockout (KO) rodent models and antisense oligonucleotide-mediated silencing confirm the pathogenic relevance of these receptors. Studies using mouse chimera models revealed that TLR2 located within the renal parenchyma is the primary driver of tissue damage [64]. This intrinsic injury mechanism is specific to the kidney and differs from observations in myocardial infarction, where infiltrating neutrophils and monocytes are the primary effectors [65].

TLR2 inhibition or deficiency benefits radiation- and transplant-induced renal injury. Following KT, TLR2-deficient (TLR2-/-) mice or those treated with antisense oligonucleotides show reduced tubular epithelial apoptosis, decreased cellular infiltration, and clear functional and structural protection [65]. Likewise, TLR4 deficiency or MyD88 deficiency protects against renal injury following I/R, a finding that correlates with the post-ischemic release of endogenous TLR2/4 ligands such as HMGB1, hyaluronan, and brevican [66].

Interestingly, TLR2-deficient models (TLR2-/-) exhibit superior renal preservation compared to MyD88-deficient models. This suggests that tissue damage involves TLR2-driven signaling pathways that are independent of the classical MyD88 cascade [67].

Comparative analysis of mice with single TLR2-/- or TLR4-/- vs. double TLR2/4-/- gene inactivation showed consistent renoprotection with the individual deletions; however, the combined disruption of both receptors did not yield a cumulative or synergistic benefit [68]. This functional overlap suggests the presence of crosstalk or co-activation between TLR2 and TLR4 upon contact with endogenous ligands released during reperfusion [69].

Despite advances, the TLR network in I/R pathology presents paradoxes that remain unclear. Although deleting TLR2 or TLR4 alone preserves function and reduces inflammation, deleting both does not enhance protection. The exact roles of TLRs and the prevalence of MyD88-dependent and -independent pathways vary by injured tissue and clinical manifestation [70,71].

DAMPs are postulated to act as endogenous agonists that trigger sterile inflammation and acute kidney injury (AKI). Furthermore, TLR2 signaling modulates immunological acceptance of the graft and plays a critical role in chronic renal allograft dysfunction [69]. However, emerging therapeutic strategies such as SGLT2 inhibitors modulate the TLR network during cold or warm ischemia. By mitigating the impact of DAMPs on TLR2 and TLR4, these drugs reduce the incidence of acute tubular necrosis, preserve mitochondrial integrity, and prevent progression of AKI to CKD. These findings provide preliminary support for developing therapies targeting TLR2; however, extensive research is required to fully elucidate these mechanisms and to evaluate the clinical efficacy of TLR2 and TLR4 antagonists across various I/R pathologies [70,71,72].

### 2.4. Role of TLRs in KT

From a topological perspective, TLRs exhibit specific subcellular localizations adapted to the nature of their ligands; plasma membrane TLRs: TLR1, TLR2, TLR4, TLR5, TLR6, and TLR11 are anchored to the cell surface, enabling them to detect extracellular signals and molecular patterns exposed in the external environment. In contrast, TLR3, TLR7, TLR8, TLR9, and TLR10 reside in endosomal and lysosomal compartments; they specialize in recognizing nucleic acids—derived from both internalized pathogens and damaged host tissue—after endocytosis and processing [73]. In KT, TLR2 and TLR4 act as the primary sensors of innate immunity. These receptors are predominantly expressed on proximal tubular epithelial cells (TECs), microvascular endothelial cells, and resident myeloid populations (macrophages and DCs). Their primary function is to detect endogenous DAMPs (HMGB1, heat shock proteins, and cell-free mitochondrial DNA) released in response to various clinical insults. Cold and warm I/R injury occurs during donor organ retrieval, preservation, and implantation [74]. SGLT2 inhibitors emerge as key modulators by suppressing TLR expression and signaling pathways within the graft, acting through metabolic and immunomodulatory pathways [74].

### 2.5. TLR2 in KT

Mapped to locus 4q32 on human chromosome 4, the gene encoding TLR2 is predominantly expressed on the membranes of circulating leukocytes. Upon binding bacterial motifs or endogenous ligands, TLR2 initiates signal transduction cascades that activate key transcription factors, including NFkB and AP-1 [75]. Following ischemic injury, massive apoptosis in target tissues triggers downstream immune programs that govern both tissue repair and host defense [76]. However, excessive leukocyte recruitment, along with the release of pro-inflammatory mediators, often drives secondary tissue destruction.

In kidney I/R injury, TLR2 signaling modulates this inflammatory balance, and receptor dysregulation or ablation directly influences leukocyte infiltration, tubular epithelial damage, and overall renal functional decline. Proteinuria may result from inflammatory processes and DAMPs that induce tubulointerstitial inflammation by activating the TLR2-MyD88-NF-κB pathway [77].

To exert its broad ligand-binding capacity, TLR2 forms homodimers or heterodimers with TLR1 or TLR6, triggering pro-inflammatory cascades via canonical MyD88-dependent signaling. Through these conformational arrangements, TLR2 senses a wide array of foreign microbial motifs and host-derived danger signals [78]. Key endogenous agonists liberated during cellular stress or necrotic damage include lipopeptides, HMGB1, heat shock protein 60 (HSP60), and HSP70, as well as extracellular matrix degradation fragments such as biglycan. Upon ligand engagement, TLR2 signaling drives NFkB nuclear translocation, prompting the secretion of critical pro-inflammatory cytokines (TNF-α and IL-6) across both parenchymal and immune cell populations. Within the renal microenvironment, TLR2 is constitutively expressed in glomerular endothelial and tubular epithelial cells. Notably, proteinuric stress induces tubulointerstitial inflammation by triggering the TLR2–MyD88–NFkB signaling axis, culminating in sustained local production of TNF-α and IL-6 [79].

However, the specific mechanism by which the TLR2 gene acts on proteinuria after KT still requires further investigation [80]. Proteinuria following KT stems from heterogeneous clinical factors that synergistically drive allograft dysfunction and adversely impact graft longevity. Systematic monitoring of urinary protein excretion, alongside prompt histological evaluation, facilitates early identification of graft pathology and permits tailored treatment strategies to preserve long-term KT function [81].

TLR2 functions in tandem with TLR1 or TLR6 to recognize Gram-positive bacterial components, whereas LPS serves as the prototypical exogenous ligand for TLR4 [82]. Signaling through TLR4 strictly requires the coreceptor CD14, which captures LPS and presents it to the TLR4–myeloid differentiation factor 2 (MD-2) complex; this interaction triggers downstream cascades that upregulate pro-inflammatory cytokines such as IL-1β and TNF-α. Despite these well-defined pathways, the clinical impact of genetic polymorphisms in TLRs and CD14 on acute transplant rejection remains controversial [83]. Nevertheless, substantial translational evidence shows that post-transplant I/R injury in kidney and lung grafts upregulates TLR2 and TLR4 mRNA expression in tubular epithelium, creating a pro-inflammatory microenvironment that fuels allograft rejection [84].

Both TLR2 and TLR4 are essential mediators of renal injury across diverse etiologies, including I/R insults, cisplatin nephrotoxicity, glomerulonephritis, and graft rejection [85]. Animal models of STZ-induced diabetes show heightened TLR2 and TLR4 expression, along with activation of their downstream cascades [86]. In diabetic patients, increased TLR2/4 activity strongly correlates with microvascular damage, highlighting the role of TLR2/4 in human diabetic nephropathy [87]. Mechanistically, TLR4 exacerbates tubulointerstitial inflammation, while TLR2 deficiency mitigates renal damage by decreasing albuminuria, podocyte loss, macrophage infiltration, and pro-inflammatory signaling [88]. Notably, TLR activation extends to glomerular podocytes, where endogenous TLR4 ligands drive injury in membranoproliferative glomerulonephritis [89]. Finally, sustained hyperglycemia induces TLR2 expression in human monocytes, increasing receptor expression and circulating ligand concentrations in type 2 diabetes [90].

The relationship between SGLT2 inhibitors and TLR2 in the KT centers on the anti-inflammatory and immunomodulatory effects of these drugs regarding graft protection. Although the natriuretic and hemodynamic mechanisms of SGLT2 inhibitors are best known, their interactions with the TLR2 and TLR4 inflammatory signaling pathways also play a significant role. The TLR2/NF-kappaB pathway helps prevent interstitial fibrosis and tubular atrophy (IFTA), a leading cause of late graft loss [91].

### 2.6. TLR4 in KT

As a key cell-surface PRR, TLR4 is involved in the pathogenesis of various infectious, autoimmune, and sterile inflammatory conditions. Its broad binding spectrum allows it to sense both exogenous PAMPs (LPS) and host DAMPs released during cellular stress, necrosis, or macromolecular breakdown. In KT TLR4-driven detrimental immune responses pose a major challenge to graft survival [92]. Additionally, TLR4 has been implicated in radiation-induced kidney injury (RIK) within renal grafts.

I/R injury induces marked TLR4 overexpression across parenchymal structures as well as infiltrating immune cells. During I/R, the massive release of endogenous DAMPs (such as HMGB1) engages the TLR4 axis; in turn, it stimulates secretion of key inflammatory mediators, facilitates leukocyte extravasation, and bridges innate and adaptive immune responses. The graft injury aggravates acute inflammation, accelerates renal fibrogenesis, and contributes to delayed graft function (DGF) and allograft rejection. Sustained TLR4 (and TLR2) overexpression marks subclinical inflammation during the critical first post-transplantation year [93]. Human kidney biopsies confirm significantly higher TLR4 expression in patients with chronic rejection than in stable controls. Beyond tissue expression, molecular profiling of peripheral blood leukocytes reveals that TLR4 and its canonical downstream adapter, MyD88, serve as reliable biomarkers for differentiating patients with chronic rejection from operationally tolerant recipients [94].

Pharmacological inhibition of TLR4 represents a promising strategy to attenuate I/R injury, reduce DGF, and enhance long-term allograft survival [95]. In this context, SGLT2 inhibitors offer crucial non-hemodynamic renoprotection by directly targeting the TLR4 axis [96]. SGLT2 inhibitors downregulate TLR4 gene and protein expression in renal tissue [97]. By inhibiting downstream MyD88-dependent signaling, they block nuclear translocation of NF-kB, drastically reducing pro-inflammatory cytokine production (IL-1β, IL-6, and TNF-α) in TECs [98]. Downregulation of TLR4, DAMPs, neutrophil, and M1 macrophage recruitment into the graft while curbing pyroptosis in proximal tubule cells [99]. SGLT2 inhibitors reduce key profibrotic markers (TGF-β1, α-SMA, and type I collagen), preventing the progression of parenchymal graft fibrosis [100].

## 3. Management Alternatives in KT

Pharmacological management in KT relies on combination protocols that target complementary immune mechanisms. Standard maintenance regimens integrate calcineurin inhibitors (CNIs); tacrolimus or cyclosporine A (CsA), mTOR inhibitors (everolimus, sirolimus), antiproliferative agents (mycophenolate derivatives, azathioprine), corticosteroids, and biological agents for induction or maintenance. Because each drug presents a unique toxicity profile and interaction spectrum, régimens must be individualized according to the recipient’s baseline risk, comorbidities, and rejection profile. However, next-generation immunosuppressive therapies remain urgently needed to minimize toxicity and prolong long-term graft survival [101].

Post-transplant hypertension is heavily influenced by the choice of immunosuppressants, necessitating tailored antihypertensive strategies. Glucocorticoids elevate blood pressure primarily via volume retention and renin–angiotensin–aldosterone system (RAAS) stimulation. CNIs induce hypertension through renal vasoconstriction and enhanced sodium reabsorption, with CsA exhibiting more pronounced hypertensive effects than tacrolimus [102]. Conversely, purine synthesis inhibitors (mycophenolate mofetil, azathioprine) and mTOR inhibitors remain hemodynamically neutral. Dihydropyridine calcium channel blockers (CCBs) are preferred during the early post-transplant phase because they directly counteract CNI-induced renal vasoconstriction. Meta-analytic evidence shows that CCBs reduce the risk of graft loss by 42% and improve glomerular filtration rate (GFR) by 11.11 mL/min compared to ACE inhibitors or ARBs. Additionally, thiazide and thiazide-like diuretics effectively mitigate CsA-mediated sodium retention. Surgical reperfusion following ischemia triggers acute sterile inflammation, causing tissue injury and the massive release of sequestered DAMPs. PRRs, particularly TLRs, sense these endogenous danger signals and initiate acute inflammatory cascades. Because the innate and adaptive immune branches are functionally interdependent, early TLR activation during I/R injury directly shapes the downstream adaptive immune responses that drive both acute and chronic allograft rejection [103].

### 3.1. Immunosuppressive Drugs and Biologics in KT

Biological agents, particularly targeted antibodies, represent cornerstone components of induction regimens and anti-rejection protocols in solid organ transplantation. Induction strategies aim to inhibit lymphocyte activation, proliferation, or survival, thereby attenuating early alloimmune responses [104]. Standard induction agents in KT include basiliximab, alemtuzumab, and antithymocyte globulin (ATG) [105].

Basiliximab is a chimeric anti-CD25 monoclonal antibody that selectively blocks the interleukin-2 receptor α-chain (IL-2R\α), preventing downstream IL-2-driven T-cell proliferation without causing profound lymphocyte depletion.

A humanized monoclonal antibody targeting CD52, inducing widespread lysis of T and B lymphocytes, monocytes, and natural killer (NK) cells. This results in profound, sustained T-cell depletion alongside temporary reductions in other immune subsets [105].

Antithymocyte Globulin (ATG) is a polyclonal antibody preparation that offers broader immunosuppressive coverage than monoclonal therapies by targeting multiple surface antigens across various lymphocyte populations [106].

Rituximab is a monoclonal antibody targeting CD20 to deplete B-cell lineages. It is effectively utilized to reduce relapse rates in steroid-dependent or frequently relapsing nephrotic syndrome; however, its therapeutic efficacy in recurrent focal segmental glomerulosclerosis (FSGS) post-KT remains controversial [107]. Eculizumab and Belatacept are used to manage complement-mediated microangiopathies and to provide CNI-free costimulation blockade, respectively [108].

From a pharmacokinetic perspective, co-administering SGLT2 inhibitors (dapagliflozin, empagliflozin, or canagliflozin) alongside maintenance immunosuppressive drugs displays no clinically relevant adverse drug–drug interactions [109]. Adding SGLT2 inhibitors does not alter trough plasma levels of baseline immunosuppressants (CNIs or mTOR inhibitors), nor does it increase the incidence of acute allograft rejection. Pharmacodynamically, their combined use yields significant nephroprotective synergy by mitigating metabolic and hemodynamic stress, while requiring standard clinical precautions for volume status and euglycemic ketoacidosis [110].

### 3.2. Calcineurin Inhibitors

CsA and tacrolimus form the cornerstone of post-transplant immunosuppressive protocols. CsA, a neutral cyclic peptide of eleven amino acids isolated from *Tolypocladium inflatum*, binds intracellularly to cyclophilin. This immunophilin complex inhibits calcineurin phosphatase activity, preventing the nuclear translocation of nuclear factor of activated T-cells (NFAT). Beyond suppressing NFAT-dependent signaling, CsA inhibits transcription factors such as NF-kB, thereby halting the gene expression of essential T-cell cytokines, including interleukin-2 (IL-2). CsA undergoes extensive hepatic and intestinal metabolism via the cytochrome P450 (CYP3A) system, generating metabolites with significantly lower immunosuppressive potency than the parent compound [111].

Despite their potent immunosuppressive efficacy, chronic CNI administration induces renal arteriolar vasoconstriction, driving chronic allograft injury, interstitial fibrosis, and tubular atrophy (IFTA) [112]. CNIs cause persistent afferent arteriolar constriction, increasing intraglomerular pressure and metabolic stress.

Co-administering SGLT2 inhibitors directly mitigates this toxic pathway by reducing tubular hyperfiltration and metabolic workload, exerting a synergistic renoprotective effect that counteracts CNI-induced chronic injury and IFTA. Tacrolimus therapy significantly increases the incidence of post-transplant diabetes mellitus (PTDM). Integrating SGLT2 inhibitors into maintenance regimens provides effective glycemic control and promotes body weight reduction without increasing the risk of hypoglycemia [113].

### 3.3. Tacrolimus and Dyslipidemia Management

Tacrolimus and CsA serve as cornerstone maintenance immunosuppressants in solid organ transplantation, typically combined with baseline biological induction agents (IL-2RAs, alemtuzumab, or antithymocyte globulin) and specialized biologics (e.g., rituximab, belatacept, eculizumab) [114,115,116,117,118].

Despite their potent efficacy in preventing graft rejection, calcineurin CNIs induce post-transplant dyslipidemia by driving hepatic low-density lipoprotein cholesterol (LDL-C) synthesis and impairing peripheral triglyceride clearance. CsA exerts dose-dependent, atherogenic effects on lipid metabolism. It downregulates hepatic LDL receptor expression by upregulating PCSK9 and impairs intestinal cholesterol clearance by inhibiting cholesterol 7-α-hydroxylase. Tacrolimus has a distinctly more favorable metabolic profile than CsA. While tacrolimus can cause mild LDL-C elevations, it produces much less pronounced lipid changes. Consequently, converting patients from CsA to tacrolimus leads to a clinically meaningful reduction in total circulating lipid concentrations [119].

Integrating SGLT2 inhibitors into tacrolimus- or CsA-based regimens does not introduce adverse drug–drug interactions or alter immunosuppressive drug exposure [120]. Tacrolimus and CsA undergo extensive first-pass and hepatic metabolism mediated by cytochrome P450 3A4 (CYP3A4) and the P-glycoprotein (P-gp) efflux transporter. In contrast, SGLT2 inhibitors are cleared primarily via uridine 5′-diphospho-glucuronosyltransferase (UGT)- mediated glucuronidation. This divergence in enzymatic degradation prevents metabolic competition in hepatic and intestinal compartments, ensuring stable trough concentrations of tacrolimus and CsA without increasing the risk of therapeutic failure or acute allograft rejection [121].

### 3.4. Mammalian Target of Rapamycin (mTOR) Inhibitors

Mammalian target of rapamycin (mTOR) inhibitors share a unified mechanism of action that arrests lymphocyte activation and antibody synthesis while curbing non-immune cellular proliferation, including fibroblasts, endothelial cells, and smooth muscle cells. After oral administration, mTOR inhibitors are rapidly absorbed; however, their systemic bioavailability varies significantly because of mucosal permeability, dietary intake, P-glycoprotein-mediated intestinal efflux, and extensive hepatic CYP3A4 metabolism. Despite their antiproliferative advantages and protective effects on long-term allograft function when combined with lower-dose CNIs, mTOR inhibitors exhibit a distinct adverse-effect profile [122].

Severe drug-induced toxicities, such as interstitial pneumonitis, can develop via T-cell-mediated delayed-type hypersensitivity or autoimmune reactivity against latent antigens. Managing this complication typically necessitates drug discontinuation and therapeutic substitution. Dyslipidemia is one of the most prominent, dose-dependent metabolic side effects of mTOR inhibitors [123]. They markedly elevate total cholesterol, LDL-C, non-HDL cholesterol, VLDL, and triglycerides by increasing hepatic apoB100 synthesis and VLDL-C secretion while decreasing PCSK9-mediated hepatic LDL-C catabolism [123]. These lipid alterations improve with dose reduction or lower trough levels; notably, no direct clinical trials currently demonstrate superior lipid safety with everolimus compared with sirolimus [124].

Co-administration of SGLT2 inhibitors and mTOR inhibitors is clinically relevant because of their metabolic and renoprotective overlap. SGLT2 inhibitors do not alter the systemic exposure, metabolism, or blood trough concentrations of everolimus or sirolimus. Pharmacodynamically, SGLT2 inhibitors directly counteract the metabolic and renal complications associated with mTOR inhibitors. They mitigate drug-induced hypertriglyceridemia and hyperglycemia, reduce intraglomerular pressure, and attenuate proteinuria, providing significant overall allograft protection [125].

### 3.5. Mycophenolic Acid and Antiproliferative Agents

As essential antimetabolite agents in KT, mycophenolic acid (MPA) and its clinically deployed formulations (mycophenolate mofetil (MMF) and enteric-coated mycophenolate sodium (EC-MPS)) prevent allograft rejection by halting lymphocyte expansion. MPA functions as a potent, selective, and reversible inhibitor of inosine monophosphate dehydrogenase (IMPDH), disrupting *de novo* guanine nucleotide synthesis and downstream DNA replication. Because T- and B-cell lineages rely predominantly on this pathway rather than the salvage pathway, MPA selectively inhibits lymphocyte proliferation. Additionally, MPA suppresses protein glycosylation in lymphocytes and monocytes, impairing their endothelial adhesion molecules and curbing recruitment into the allograft parenchyma. Unlike calcineurin inhibitors and mTOR inhibitors, antiproliferative agents and biological induction therapies display a remarkably neutral lipid profile. MMF, enteric-coated MPA, azathioprine, and biological induction agents (such as polyclonal anti-lymphocyte antibodies, anti-CD25 monoclonal antibodies, and rituximab) do not alter circulating lipid concentrations or induce dyslipidemia [126].

Co-administering MPA formulations with SGLT2 inhibitors (dapagliflozin, empagliflozin, and canagliflozin) demonstrates complete pharmacological compatibility. No detrimental pharmacokinetic interaction or direct contraindication exists between MPA formulations (MMF/EC-MPS) and SGLT2 inhibitors in KT recipients.

SGLT2 inhibitors and MPA do not alter each other’s trough blood concentrations, nor do they compete in a clinically relevant manner regarding gastrointestinal absorption, glucuronidation, or renal elimination [127].

### 3.6. Glucocorticoids

Corticosteroids provide essential immunosuppressive, anti-inflammatory, and immunomodulatory coverage to prevent allograft rejection, although steroid-sparing and withdrawal protocols are increasingly implemented in stable, low-risk recipients [128]. Despite their therapeutic efficacy, chronic exposure to prednisone drives substantial systemic toxicity across multiple organ systems, including the kidneys [129].

Because no single regimen fits all clinical scenarios, modern immunosuppression relies on individualized multi-drug combinations tailored to recipient risk profiles and institutional guidelines. Regimen selection and induction choices depend on donor type (living vs. deceased), HLA matching, organ cold ischemia time, recipient immunological risk, baseline comorbidities, and infection history. Current standard maintenance regimens center on triple-drug therapy: a calcineurin inhibitor (CNI), an antiproliferative antimetabolite, and corticosteroids. Glucocorticoids induce pronounced, dose-dependent metabolic and lipid alterations [130]. Corticosteroids enhance hepatic VLDL synthesis and downregulate hepatic LDL receptor expression, likely via glucocorticoid receptor-mediated transcriptional modulation. This mechanism drives dose-dependent elevations in total circulating cholesterol, LDL-C, VLDL, and triglycerides (TG) [131].

The clinical rationale for co-administering SGLT2 inhibitors alongside corticosteroid-based immunosuppression rests on metabolic counter-regulation and complementary renal hemodynamic protection [132]. Corticosteroids do not alter the metabolic clearance or systemic exposure of gliflozins, nor do SGLT2 inhibitors affect corticosteroid pharmacokinetics [133].

Both corticosteroids and CNIs alter glomerular hemodynamics. SGLT2 inhibitors restore tubuloglomerular feedback (TGF), decreasing hyperfiltration, intraglomerular pressure, and allograft proteinuria [134]. By improving urinary glucose excretion and insulin sensitivity, SGLT2 inhibitors directly counteract corticosteroid-induced post-transplant diabetes mellitus (PTDM), weight gain, and systemic dyslipidemia, thereby preserving long-term graft function [135].

### 3.7. Other Management Alternatives in RT

#### 3.7.1. Sodium-Glucose Cotransporter 2 Inhibitors

In April 2021, dapagliflozin became the first SGLT2 inhibitor approved by the FDA for CKD, regardless of diabetes status. Originally developed for glycemic control, SGLT2 inhibitors are now recognized as foundational therapies in renal care, offering robust nephroprotection across diverse CKD etiologies [136].

SGLT2 inhibitors induce glycosuria, osmotic diuresis, and natriuresis by blunting glucose and sodium reabsorption in the early nephron [123]. This increased sodium delivery to the macula densa activates tubuloglomerular feedback, reducing intraglomerular pressure and mitigating hyperfiltration [137]. Systematically, these hemodynamic shifts translate into modest weight loss, extracellular volume contraction, and sustained reductions in blood pressure [138].

Beyond hemodynamics, SGLT2 inhibitors attenuate metabolic and cellular pathways driving disease progression [130]. Decreased tubular transport reduces proximal metabolic workload and oxygen consumption, alleviating cortical hypoxia and subsequent tubulointerstitial fibrosis [139]. At the cellular level, SGLT2 inhibitors optimize mitochondrial function, limit ROS production, and promote mild ketosis [140]. Furthermore, SGLT2 inhibition suppresses key pro-inflammatory cytokines (IL-6 and TNF-α) and blunts TGF-β1-driven fibrotic signaling [141].

In KT recipients, particularly those with post-transplant diabetes mellitus, SGLT2 inhibitors improve glycemic control (HbA1c) and support weight management, with the greatest clinical efficacy observed in patients with preserved eGFR [142].

#### 3.7.2. SGLT2 Inhibitors in KT (Dapagliflozin in KT)

Although SGLT2 inhibitors offer significant therapeutic potential in KT, clinical evidence supporting their routine use remains limited. Early safety concerns led to the systematic exclusion of transplant recipients from pivotal trials. Consequently, clinicians continue to weigh their potential graft and cardiovascular benefits against a developing safety profile [143].

While RAAS inhibitors were long the sole disease-modifying therapy for proteinuric kidney disease, SGLT2 inhibitors have established a new standard of care in native CKD and heart failure. In the transplant setting, their pleiotropic actions offer substantial promise for managing recipients with diabetes, proteinuria, or heart failure—potentially improving long-term allograft survival and mitigating cardiovascular morbidity [144].

However, definitive long-term outcomes have yet to be established in robust transplant cohorts. Existing literature consists primarily of short-term observational studies characterized by methodological heterogeneity, variable follow-up durations, and non-standardized endpoints, complicating cross-study synthesis and limiting definitive clinical guidance [145].

#### 3.7.3. Mechanisms of SGLT2 Inhibitor Efficacy and Potential Benefits in KT

**Figure 6:**
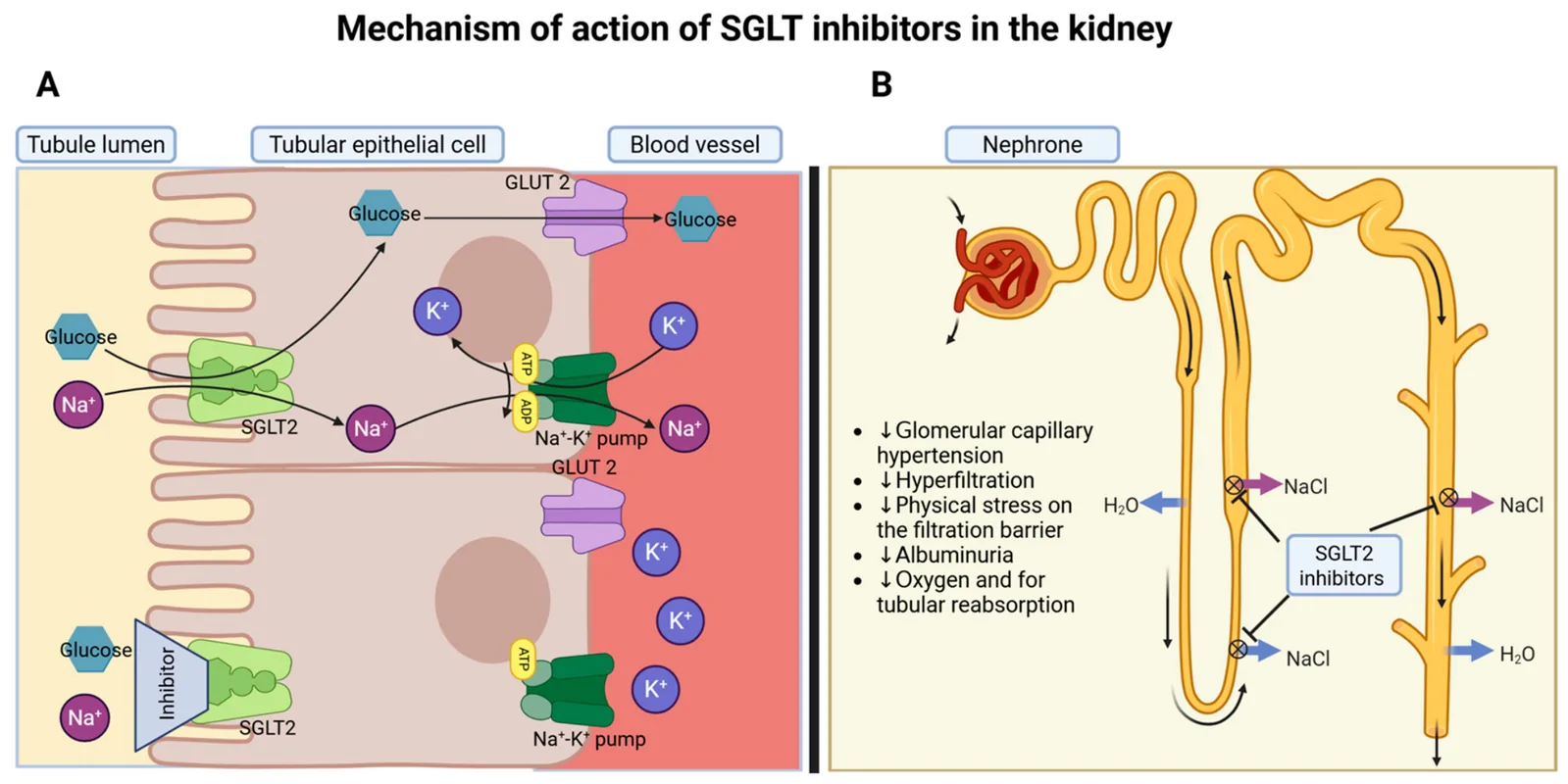
Mechanism of action of SGLT2 inhibitors in the kidney. (**A**) Sodium-glucose cotransporter (SGLT) proteins facilitate the uptake of glucose and sodium into tubular epithelial cells; subsequently, glucose enters the circulation via passive transport, while sodium does so through active transport mediated by the sodium-potassium pump. SGLT2 inhibitors block Na+- glucose cotransport. (**B**) Blocking SGLT2 increases the delivery of sodium, chloride, and glucose to the distal tubule. Through this mechanism, SGLT2 inhibitors decrease glomerular capillary hypertension and hyperfiltration, thereby reducing physical stress on the filtration barrier, albuminuria, and the oxygen demand for tubular reabsorption [156]. Created in BioRender. Garcia, A. (2026) https://BioRender.com/8kqpil1 (accessed on 17 August 2026).

Inhibition of SGLT2-mediated co-transport in the S1 segment of the proximal tubule increases distal delivery of sodium and chloride, activating tubuloglomerular feedback via the macula densa. This induces afferent arteriolar constriction, relieving intraglomerular hypertension and mitigating hyperfiltration (Figure 6). Clinically, this mechanism manifests as an initial, reversible dip in eGFR (approximately 5 mL/min/1.73 m^2^) before renal function stabilizes [146]. Unlike RAAS, SGLT2 inhibitors protect structural integrity by concurrently lowering filtration barrier stress, urinary protein excretion, and metabolic demand [147,148]. Reducing post-transplant proteinuria is particularly critical, as it remains a strong predictor of allograft failure, post-transplant diabetes mellitus (PTDM), and cardiovascular mortality [149].

By blunting solute reabsorption, SGLT2 inhibitors lower the proximal tubular workload and epithelial oxygen consumption. This energetic relief preserves tubular integrity against ischemic injury and prevents chronic allograft dysfunction [150]. Additionally, SGLT2 inhibition promotes a substrate shift toward lipid metabolism and mild ketosis, providing renal epithelial cells and cardiomyocytes with a bioenergetically efficient fuel source that mitigates ROS production and alleviates cellular stress [151].

The glycemic efficacy of SGLT2 inhibitors depends directly on filtration capacity: HbA1c reductions are modest in advanced CKD (ranging from 0.7% when eGFR >60 mL/min/1.73 m^2^ to 0.3–0.4% when eGFR is 45–60 mL/min/1.73 m^2^) [152]. Consequently, their benefits in advanced stages of renal disease stem primarily from non-glycemic pathways. In transplant recipients, however, even modest glucose lowering reduces β-cell stress and improves peripheral insulin sensitivity, making SGLT2 inhibitors valuable adjuncts for preventing and managing PTDM [153].

Post-transplant hypertension affects 50–80% of recipients, largely driven by immunosuppressive regimens (CNI and glucocorticoids) that promote renal sodium retention and vasoconstriction [154]. SGLT2 inhibitors induce sustained natriuresis and osmotic diuresis, reducing systolic blood pressure by 4–10 mmHg without triggering compensatory RAAS activation [155]. Notably, dapagliflozin preferentially clears interstitial fluid rather than intravascular volume, thereby providing effective decongestion while optimizing ventricular loading conditions—a mechanism vital for mitigating cardiovascular risk in transplant recipients [156].

#### 3.7.4. Influence of SGLT2 Inhibitors on Innate and Adaptive Immunity

Beyond glycemic and hemodynamic effects, SGLT2 inhibitors exert significant anti-inflammatory effects by modulating nutrient-sensing pathways (AMPK, sirtuins, mTOR) and blunting low-grade metabolic inflammation [157]. In innate immune cells, SGLT2 inhibitors suppress the TLR4/NF-κB, MAPK, and JAK/STAT pathways, independently of ambient glucose levels [158]. In human monocyte-derived macrophages, dapagliflozin attenuates LPS-induced TLR4 expression, restores anti-inflammatory miR-146a, represses pro-inflammatory miR-155, and promotes a favorable shift toward the M2 anti-inflammatory macrophage phenotype [159].

SGLT2 inhibitors directly inhibit the NLRP3 inflammasome—a central driver of caspase-1-dependent processing of pro-IL-1β and pro-IL-18 into active inflammatory cytokines [160]. Mechanistically, dapagliflozin increases circulating levels of itaconate, an endogenous metabolite that inhibits NLRP3 activation. By suppressing this axis alongside NF-κB signaling, SGLT2 inhibitors significantly reduce systemic and intrarenal levels of TNF-α, IL-6, and IL-1β [161]. Additionally, SGLT2 inhibition modulates non-classical RAAS pathways and dampens intrarenal sympathetic activation, further blunting inflammation and fibrosis [162].

In the renal parenchyma and vasculature, SGLT2 inhibitors preserve endothelial viability and prevent osteogenic transdifferentiation of vascular smooth muscle cells (VSMCs) [163]. Agents like canagliflozin and dapagliflozin limit vascular calcification by repressing osteogenic factors (RUNX2, BMP2) and downregulating endoplasmic reticulum stress proteins such as TXNDC5 [164]. In proximal tubular epithelial cells, dapagliflozin mitigates oxidative stress and represses the HMGB1/RAGE/NF-κB and YAP/TAZ signaling axes [165]. This suppresses downstream fibrotic mediators, thereby delaying tubulointerstitial fibrosis [166]. High-throughput single-cell transcriptomics and omics profiling continue to map these epigenetic and cellular responses across immune sublineages [167].

Although clinical trials in renal allograft recipients are constrained by smaller cohort sizes and shorter follow-up periods compared with native CKD populations, the biological plausibility of protection is compelling [168]. By concurrently alleviating intraglomerular pressure, reducing tubular metabolic workload, and suppressing pro-inflammatory and pro-fibrotic signaling (NF-κB, YAP/TAZ), SGLT2 inhibitors offer a multifaceted defense against the dual challenges of chronic allograft dysfunction and premature graft failure.

### 3.8. Complications Associated with Immunosuppressive Therapy

Although essential for preventing allograft rejection, long-term immunosuppressive regimens induce widespread multi-organ toxicities that drive post-transplant morbidity and mortality [169].

Immunosuppressive agents frequently induce post-transplant diabetes mellitus, hypertension, and hyperlipidemia, providing strong rationale for early steroid-minimization protocols. Comprehensive cardiovascular management requires routine screening and correction of post-transplant anemia, alongside targeted supplementation with vitamin D, folic acid, and vitamins B6 and B12. Structured lifestyle interventions like smoking cessation, nutritional counseling, and regular exercise are equally essential to mitigate cardiovascular mortality [170].

Cancer represents the second leading cause of death in renal transplant recipients, driven primarily by non-melanoma skin cancers and renal cell carcinoma (RCC). Notably, transplant recipients have a significantly higher incidence of RCC than the general population (approximately 0.1%) [171].

Cytomegalovirus (CMV) remains a formidable challenge, with clinical risk governed chiefly by donor/recipient pre-transplant serostatus. The choice of maintenance immunosuppression heavily modulates this risk; for example, belatacept therapy is associated with increased susceptibility to primary CMV infection and prolonged viremia in high-risk patients [172].

Urinary tract infections (UTIs) are highly prevalent within the first 12 months post-transplant, with reported incidence rates ranging from 7% to 80%. Up to 90% of these cases are caused by Gram-negative bacilli, predominantly *Escherichia coli*. Furthermore, hospitalized solid organ transplant recipients face a five-fold higher risk of acquiring *Clostridioides difficile* infection compared to non-immunocompromised controls [173].

## 4. Conclusions

Long-term success in KT remains fundamentally constrained by a complex continuum of tissue injury and immune activation. From the initial insult of IRI during organ procurement to the chronic, insidious drivers of DGF, CNI toxicity, and chronic allograft dysfunction, sterile inflammation stands as the ultimate arbiter of graft survival. While conventional maintenance immunosuppression successfully prevents acute adaptive rejection, it leaves non-immune, metabolic, and innate inflammatory pathways largely unaddressed—frequently at the cost of severe cardiovascular, metabolic, and infectious toxicities.

SGLT2 inhibitors represent a paradigm shift in post-transplant management. Beyond their original intent as glucosuric agents, SGLT2 inhibitors function as multifaceted immunomodulatory and cytoprotective therapies that work synergistically with standard immunosuppressive regimens. By targeting core cellular, bioenergetic, and inflammatory networks, SGLT2 inhibitors provide comprehensive protection against graft failure. By activating tubuloglomerular feedback via the macula densa, SGLT2 inhibitors reduce intraglomerular pressure, mitigate hyperfiltration, and alleviate metabolic strain on proximal tubular epithelial cells. This reduced active-transport workload preserves intracellular ATP, attenuates cortical hypoxia, and shields vulnerable tubular tissue from ischemic injury during I/R injury.

SGLT2 inhibitors suppress the surface expression and gene transcription of key PRR tubular, endothelial, and resident myeloid lineages. By reducing necrotic cell death and preserving mitochondrial membrane integrity, these agents limit the extracellular release of endogenous alarmins (DAMPs such as HMGB1 and heat shock proteins), thereby breaking the self-perpetuating cycle of sterile inflammation.

At the intracellular level, SGLT2 inhibition activates the AMP-activated protein kinase (AMPK) pathway and represses MyD88-dependent NF-κB signaling. This dual action halts both the priming and assembly of the NLRP3 inflammasome, blunting the release of pro-inflammatory cytokines (IL-1β, IL-18, TNF-α, and IL-6). Furthermore, by repressing TGF-β1, CTGF, and the YAP/TAZ signals, SGLT2 inhibitors directly counteract interstitial fibrosis and tubular atrophy (IFTA).

SGLT2 inhibitors actively shape the intragraft leukocyte microenvironment. By downregulating chemoattractants (MCP-1/CCL2) and endothelial adhesion molecules (ICAM-1, VCAM-1), they restrict inflammatory monocyte infiltration while driving a favorable phenotypic shift in macrophages from pro-inflammatory M1 effectors toward tissue-healing, anti-inflammatory M2 phenotypes.

In transplant recipients burdened by high rates of PTDM, hypertension, and fluid overload, SGLT2 inhibitors provide sustained, preferential interstitial decongestion, modest blood pressure reduction, and improved insulin sensitivity—all without inducing compensatory RAAS activation or compromised intravascular volume.

## Data Availability

No new data were created or analyzed in this study. Data sharing is not applicable to this article.
